# Influence of stigma, sociodemographic and clinical characteristics on mental health-related service use and associated costs among young people in the United Kingdom

**DOI:** 10.1007/s00787-022-01947-2

**Published:** 2022-01-27

**Authors:** Wagner Silva Ribeiro, Renee Romeo, Derek King, Shanise Owens, Petra C. Gronholm, Helen L. Fisher, Kristin R. Laurens, Sara Evans-Lacko

**Affiliations:** 1 Care Policy and Evaluation Centre, London School of Economics and Political Science, Houghton Street, London, WC2A 2AE UK; 2 Health Service and Population Research Department, Institute of Psychiatry, Psychology & Neuroscience, King’s College London, London, UK; 3 University of Washington, Seattle, WA USA; 4 Social, Genetic & Developmental Psychiatry Centre, Institute of Psychiatry, Psychology & Neuroscience, King’s College London, London, UK; 5 ESRC Centre for Society and Mental Health, King’s College London, London, UK; 6 School of Psychology and Counselling, Queensland University of Technology (QUT), Brisbane, Australia; 7 Department of Psychosis Studies, Institute of Psychiatry, Psychology & Neuroscience, King’s College London, London, UK

**Keywords:** Child and adolescent mental health, Economic costs, Service use, Stigma

## Abstract

This study examined the influence of stigma, psychopathology, and sociodemographic characteristics on mental health-related service use and costs related to service use in a cohort of young people in the UK. Using data from a community sample of young people aged 9–17 years and their caregivers, we assessed 407 young people’s use of services due to mental health problems, young people’s psychopathology, demographic characteristics, maternal education and caregivers’ stigma-related beliefs. Unit costs related to services were gathered from national annual compendia and other widely used sources. We assessed predictors of service use through logistic regression analysis and developed generalised linear models to identify factors associated with costs of mental health-related service utilisation. Persistent psychopathology, socioeconomic disadvantage, and low caregiver intended stigma-related behaviour were associated with increased likelihood of service use among young people. Older age and socioeconomic disadvantage were associated with increased costs. Different factors influenced contact with services and the cost associated with their use — persistent psychopathology and socioeconomic disadvantage increased, and caregivers’ intended stigma-related behaviour decreased the likelihood of using services, whereas socioeconomic disadvantage and older age were associated with increased costs. Social determinants of mental health problems play an important role in the use and costs of different types of mental health-related services for young people. Discordance between drivers of service use and costs implies that young people who are more likely to access services due to mental health problems do not necessarily receive care at the intensity they need.

**Supplementary Information:**

The online version contains supplementary material available at 10.1007/s00787-022-01947-2.

## Background

Mental health problems affect a significant proportion of children and adolescents worldwide [1]. In England, estimates from 2017 indicated that around one in eight children and young people had a mental disorder [2]. Although this survey reported that two-thirds of children and adolescents with mental disorders used services in the past year, a closer look at the data shows that a significant proportion of young people with mental health problems do not receive the care they need. According to a report from Children’s Commissioner [3], only “a small fraction of those who need help” in the United Kingdom (UK) accessed child and adolescent mental health services (CAMHS) in that same year: of the 338,000 children referred to CAMHS in 2017, 31% received treatment, 37% were denied treatment or discharged after the first assessment and 32% remained on waiting lists at least until the end of the year. Given that a high proportion of young people do not have access to mental health care, it is important to understand the factors that hinder their access to services.

Under-investment may be an important barrier to accessing services, as it reduces the availability of specialist and non-specialist staff, medication, and facilities. In the UK, less than 1% of the National Health Service (NHS) budget is invested in CAMHS [3]. Considering that children comprise 20% of the population, but only 9% of the overall mental health budget is invested in CAMHS, the Children’s Commissioner [3] estimated that an additional £1.7 billion per year is needed just to achieve parity between child and adult services provision. As resources are scarce, it is important, then, to make more efficient use of what is available. Therefore, it is crucial to understand which factors are associated with access to care and costs of using services.

On the demand side, there are factors other than clinical needs which may act as a barrier to access and use of services, such as socioeconomic circumstances, children’s age, gender and ethnicity [4]. Among young people, caregiver characteristics could also impede access to mental health care, as parents play an important role in deciding whether and when to seek help for their offspring [5]. Caregivers’ stigmatising beliefs about mental illness, for example, may impede recognition of problems [6], need for support, and treatment options [7, 8].

Barriers such as caregivers’ stigmatising beliefs and sociodemographic disadvantage might delay contact with services, such that young people seek services only when presenting with severe symptoms. To inform policy and practice, we examined how clinical factors and these barriers to accessing services were related to service contact and costs of mental health-related service utilisation among a cohort of young people in the UK. We tested the hypothesis that young people would have less contact with services if they were socioeconomically disadvantaged and if their parents had high intended stigma-related behaviour. We further hypothesised that the same factors that hindered contact with services would be associated with higher costs among young people who have received mental health-related care.

## Methods

### Participants

This study used data on a community sample of young people who were initially screened for mental health problems when aged between 9 and 12 years. The sample was part of the London Child Health and Development Study (CHADS), which is an ongoing prospective longitudinal investigation of children recruited through convenience sampling from collaborating primary schools in Greater London, the majority of which were located in deprived inner-city areas [6, 9–11]. In summary, during 2004–2010, 8099 children who were registered at 73 schools (*n* = 7966) or at four general practitioner’s surgeries (these surgeries participated in a pilot phase of the study only; *n* = 133) completed a mental health screening questionnaire which assessed internalising and externalising psychopathology, and psychotic-like experiences. To be included in the cohort study, they should have completed the screening questionnaire and their caregivers should have consented to participate.

**Fig. 1:**
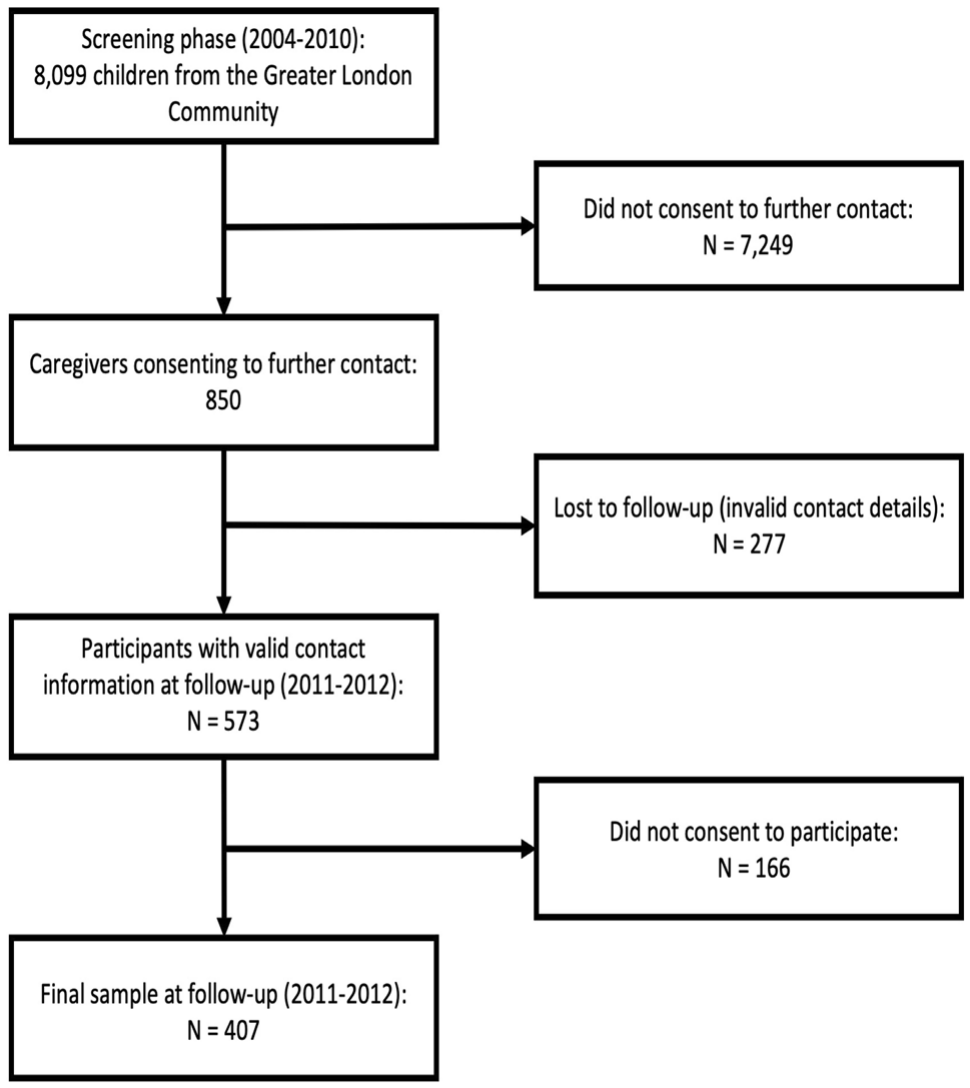
Study’s sampling flowchart

Approximately 10.5% of the young people’s primary caregivers (*n* = 850) consented to be re-contacted for further research [12]. In 2011–2012, we sought additional data from 573 children–parent dyads (67.4% of the original cohort) for whom we retained valid contact information, among whom 407 (71.0%) caregivers agreed to participate (Fig. 1 provides a flowchart of the study sampling).

A previous analysis [6], comparing the sample assessed at follow-up relative to the 443 participants without valid contact information and/or who declined participation, showed that they were similar in terms of age and gender. However, the 407 participants included in this study reported lower psychopathology scores at screening, and a greater proportion reported their ethnicity as White British or other White ethnic group relative to the 443 participants not reassessed.

### Procedure

A structured telephone interview was conducted with caregivers in which data on caregivers’ characteristics and young people’s use of mental health services were collected. These data were linked with socio-demographic information and data regarding the young people’s clinical characteristics gathered via self- and caregiver-report questionnaires at screening and follow-up [10]. The average time lapse between the first (questionnaire-based) assessment of young people’s clinical characteristics and the (interview-based) service use assessment was 2.8 years (SD = 1.4) [6]; the second questionnaire assessment of clinical characteristics was conducted contemporaneously with the service use assessment [6].

Both caregivers and young people provided written informed consent (and written assent when the young person was aged under 16 years) indicating agreement with these data collection and linkage procedures. Both the King’s College London and London School of Economics and Political Science Research Ethics Committees provided ethical approval for this study.

### Measures

Demographic characteristics: Young people’s gender (self-reported) and age (calculated from the dates of birth and assessment) were defined at the beginning of the 12-month period of follow-up data collection. Age was grouped in three categories, to reflect different stages of childhood and adolescence [13]: 9–10 years, 11–13 years, and 14 years or older. Young people’s ethnicity was reported by their caregivers according to the 2001 UK Census categories [14]. Due to the low number of participants in each of the 11 ethnic categories reported, this variable was dichotomised into White and non-White for statistical analysis. White ethnicity included White British, White Irish, and other White groups; non-White ethnicity included Black African or African Caribbean, Asian, mixed race, and other ethnic groups. Young people’s eligibility to receive free school meals, as reported by caregivers, was used as an indicator of socio-economic disadvantage [6, 15]. Mothers’ highest level of education attained was coded according to National Framework of Qualifications (NFQ) [16] levels 1 to 8. These levels were grouped into three categories: (1) *primary school* (NFQ1 and NFQ2); (2) *high school* (NFQ3 to NFQ5); and (3) *college/university* (NFQ6 to NFQ8).

Young people’s mental health problems: were assessed both at screening (2004–2010) and follow-up (2011–2012) phases using the Strengths and Difficulties Questionnaire (SDQ) [17], which includes 25 statements that reflect potential strengths and difficulties coded using three Likert-style responses (0 = “not true”; 1 = “somewhat true”; and 2 = “certainly true”). Responses to 20 of the 25 items are summed to compute four psychopathology subscales comprising five items each (Emotional Problems Symptoms; Conduct Problems; Hyperactivity-Inattention; and Peer Relationship Problems), which are then categorised as “normal”, “borderline” and “abnormal” according to UK population norms [18]. In this study, both the self- and caregiver-report versions of the SDQ were used, both of which have acceptable psychometric properties, such as satisfactory internal consistency, with Cronbach’s α varying from 0.60 to 0.85 across the SDQ subscales in the UK [17], as well as in other countries [19], and also satisfactory test–retest stability after 4–6 months (mean = 0.62) [17]. Using SDQ data from the two time-points (screening and follow-up), a variable was created that classified young people’s experience of psychopathology into four categories: (1) *no psychopathology*, defined as being classified in the “normal” range according to self- and caregiver-reports on all four psychopathology subscales at both time-points; (2) *remittent psychopathology*, defined as being classified as “borderline” or “abnormal” by either child- or caregiver-report on any subscale at screening and as “normal” by both child- and caregiver-report on all subscales at follow-up; (3) *incident psychopathology*, defined as being classified as “normal” by both child- and caregiver-report on all subscales at screening and classified as “borderline” or “abnormal” by self- or caregiver-report at follow-up; and (4) *persistent psychopathology*, defined as being classified as “borderline” or “abnormal” by either self- or caregiver-report on any subscale at both time-points.

Caregivers’ intended stigma-related behaviour: was assessed at follow-up (2011–2012) using the intended behaviour subscale of the Reported and Intended Behaviour Scale (RIBS), which is a questionnaire developed to assess the behavioural component of stigma, which has been conceptualised as comprising three dimensions [20]: (1) a knowledge dimension (ignorance), an attitude dimension (prejudice), and a behavioural dimension (discrimination). RIBS comprises two sub-scales, which assess reported (past and current) contact with, and intended behavioural acceptance or discrimination against, people with mental health problems [21]. The original RIBS scale, developed in the UK, has moderate test–retest reliability of 0.75, and good (α = 0.85) [21] to high (α = 0.95) [22] internal consistency. Similar results have been found in other languages, such as Italian [23] and Japanese, both with α = 0.83 [24], and Brazilian Portuguese (α = 0.75) [25]. The RIBS intended behaviour subscale comprises four items which assess future intentions to “live with”, “work with”, “live nearby”, and “continue a relationship with” a person with mental health problems. Items are coded using a five-level Likert scale (“agree strongly”; “agree slightly”; “neither agree nor disagree”; “disagree slightly”; and “disagree strongly”). Responses are summed into a composite score, ranging from 4 to 20, in which higher total scores reflect less intended stigmatising behaviours towards people with mental health problems. The total score was dichotomised into low (tercile 3) and medium/high (terciles 1 and 2) stigma. Terciles 1 and 2 were grouped together because the RIBS score distribution was skewed towards higher scores, with two-thirds of participants scoring between 17 and 20.

Young people’s mental health-related service use: At follow-up (2011–2012), we assessed use of services due to mental health problems in the past 12 months across different public sector organisations (health, social care, education, criminal justice) and private or voluntary service providers (such as priests, healers) and considered that some services may be outsourced and/or contracted out to a private or voluntary provider delivering the entire service on behalf of the public (such as foster care, residential treatment) using the parent-report Service Assessment for Children and Adolescents (SACA) [26]. The SACA collects information on the type, frequency, and duration of services used, and the setting in which the service was provided. The SACA demonstrates moderate to substantial agreement between caregivers’ reports and service records of children’s 12-month service use (kappa = 0.76) [27], with good past-year test–retest reliability (ranging from 0.75 to 0.86) [28]. To adapt the instrument, we carried out consultations with the developers of the original scale and with mental health specialists in the UK. Where appropriate, we replaced US services with their equivalent in the UK and supplemented it with UK-specific services – e.g., CAMHS services and 'Sure Start’ instead of Head Start.

#### Public sector service costs

For costing purposes, a public sector perspective including health, social care, education, and criminal justice services was adopted. The overall cost of care was derived by multiplying the unit of public sector services used by their unit costs. Health and Social care unit costs were obtained from a widely used national annual compendium, *Unit Costs of Health and Social Care 201*5 [29]. For services provided by the criminal justice system, costs were extracted from a 2013 publication for criminal justice services, *Unit Costs in Criminal Justice (UCCJ)* [30], and uprated to 2014/2015 prices. To uprate criminal justice services unit costs from 2008/2009 to 2014/2015, we multiplied the 2008/2009-unit cost by the arithmetic mean of the Gross Domestic Product (GDP) index numbers between 2008 and 2015. We used the GDP deflator which measures the change in price of all domestically produced goods and services, as GDP is normally used in measures of public expenditure where the focus is wider than consumer items alone. The unit costs for professionals and services not contained in the national compendium, such as teachers, teaching support staff, and special educational needs officers, were derived using salary scales published online by the National Union of Teachers [31], with add-ons for salary-related costs (e.g., pension contributions) and overheads incurred by employers. Education costs were derived using the *2014–15 Revenue Funding Arrangements* from the Education Funding Agency [32] to determine unit costs for special education. Preschool costs were derived from the Family Childcare Trust [33]. All costs were expressed in pounds sterling (£), for the 2014/15 financial year.

### Statistical analysis

We first derived prevalence information (*n*, %) for all variables to be examined in relation to the economic service costs. Next, multivariable logistic regression was used to determine which variables were associated with use of services due to mental health problems by young people in our sample. In the logistic regression analysis, the dependent variable was use of services in two categories (no vs. yes).

To assess potential predictors of costs of services that were used due to mental health problems, we applied a two-part model analysis [34] – in the first part of the model, a probit equation was used to discriminate participants who had either zero or positive costs; in the second part of the model, three Generalised Linear Models (GLM) were run to assess the relationship between independent variables and service costs only among participants who, in the first part of the model, were identified as having positive costs. In each of these models, the dependent variable was the cost of use of services due to mental health problems, expressed in pounds sterling as a continuous variable.

Model 1 included only psychopathology (categorised as none, remittent, incident and persistent) as an independent variable. Model 2 included psychopathology along with sociodemographic factors (young person’s age and gender, maternal education, and young person’s eligibility to receive free school meals). Model 3 included all variables from Model 2 and added caregivers’ intended stigmatising behaviours. In this model, we simultaneously assessed the independent effects of all three types of factors. To aid interpretation, we calculated the marginal effect on costs of each of the independent variables in Model 3. The STATA post-hoc command “mimrgns” converted the GLM coefficients into mean costs in pounds sterling (£), of each of the independent variables in Model 3.

We included the same set of independent variables in the two-part model because we hypothesised that the same variables which hinder access to mental health-related service use would be associated with higher costs, as they may lead to delayed contact with services, and thus use of more intensive and costly services once accessed.

### Handling of missing data

The psychopathological trajectory variable was missing in 10% of observations and ethnicity was missing in 1% of observations. To minimise potential selection bias due to missing values and to maximise statistical power, multiple imputation was undertaken. We assumed that these data were missing at random and imputed values using chained equations [35], in which missing values were delivered from appropriate distribution of the partially observed data. We derived ten datasets which included imputed values. The modelling results combine the estimates derived in each of these datasets, incorporating standard errors associated with the uncertainly resulting from estimation across the multiple datasets. Further, to assess the impact of the multiple imputation, we conducted a sensitivity analysis in which our primary results using the multiple imputation datasets were compared to results derived from complete-case analyses.

Participants with missing data on our psychopathology variable (*n* = 39) included a higher proportion of non-white young people (56.8% vs. 39.1%; *p* = 0.037), or young people with missing ethnicity data (5.1% vs. 0.5%; *p* = 0.006). They also included a higher proportion of young people who were eligible to receive free school meals (20.5% vs. 10.1%; *p* = 0.048). Nine participants had missing psychopathology data at both baseline and follow-up. Among the 30 participants with missing data only at the follow-up assessment, there was a higher proportion of young people presenting baseline *abnormal* psychopathology as compared to those with no missing data (33.3% vs. 15.2%; *p* = 0.010).

### Sensitivity analyses

To assess the robustness of our results, we considered in turn, the impact of using the same imputation strategy or no imputation on two different statistical models (logistic regression and GLM models) used in the analyses to identify drivers of mental health-related service use and costs.

In additional sensitivity analyses, we also explored what impact re-categorisation of the stigma and ethnicity variables would have on the results. For both the stigma and ethnicity variables, we reran the logistic regression and GLM models using alternative categorisations as follows:

1. For the stigma variable, we ran two alternative logistic regression and GLM models replacing the two-category variable first with a three-category variable in which participants were grouped into terciles, and then with the original RIBS scores as a continuous variable.
2. For ethnicity, we first reran the logistic regression and GLM models with a four-category variable (White, Black, Asian and Other), and then with an alternative two-category variable (Black vs. non-Black).

All analyses were conducted using STATA version 13.1 [36]. A significance level of 0.05 was assumed as an indicator of statistical significance.

## Results

Just over a half of our sample comprised female participants. Most participants were aged between 11 and 13 years at the follow-up assessment and reported white ethnicity. Almost half experienced persistent psychopathology and over one in ten was eligible to receive free meals at school. More than half of mothers had college/university education. Almost two-thirds of caregivers reported medium/high levels of stigma-related behaviour (Table 1). Just one in five young people used any services due to mental health problems in the past 12 months, and the mean annual cost per participant who used services was nearly £ 2,400 — we provide details on the proportion of young people using each service included in our assessment, frequency of use, unit costs and mean annual costs for each service and by sector in Supplemental Table 1.

**Table 1:** Sample characteristics (*N* = 407)

	*n*	%
Age (years)		
9–10	40	9.8
11–13	252	61.9
14 +	115	28.3
Gender		
Female	223	54.8
Male	184	45.2
Ethnicity		
White	239	59.3
Non-white	164	40.7
Missing	4	
Maternal highest level of education		
Primary school	83	20.4
High school	97	23.8
College/university	227	55.8
Young people’s eligibility for free school meals		
No	362	88.9
Yes	45	11.1
Young people’s mental health problems		
None	77	20.9
Remittent	77	20.9
Incident	41	11.1
Persistent	173	47.0
Missing	39	
Young people’s use of services		
No	323	79.4
Yes	84	20.6
Caregiver’s intended stigma-related behaviour		
Low stigma	147	36.1
Medium/high stigma	260	63.9

As can be seen in Table 2, young people were more likely to have used services if they were eligible to receive free meals at school and if their caregivers reported low levels of stigma-related behaviour.

**Table 2:** Association between use of services due to mental health problems and potential predictors based on multivariate logistic regression analysis, with random imputation of missing data (*n* = 407)

Independent variables	O.R. (95% CI)	*p* value
Young people’s mental health problems		
None	Reference	
Remittent	0.95 (0.32–2.83)	0.932
Incident	1.73 (0.54–5.60)	0.357
Persistent	2.91 (1.23–6.88)	0.015
Age (years)		
9–10	Reference	
11–13	0.67 (0.30–1.50)	0.332
14 +	0.55 (0.22–1.33)	0.185
Gender		
Female	Reference	
Male	1.43 (0.85–2.41)	0.174
Ethnicity		
White	Reference	
Non-white	1.04 (0.60–1.80)	0.882
Maternal highest level of education		
Primary school	Reference	
High school	0.68 (0.32–1.43)	0.306
College/university	0.73 (0.38 to 1.40)	0.344
Young people’s eligibility for free school meals		
No	Reference	
Yes	2.62 (1.27–4.43)	0.009
Caregivers’ intended stigma-related behaviour		
Low stigma	Reference	
Medium/high stigma	0.57 (0.07–0.93)	0.040

*CI* confidence interval, *OR* odds ratio

Table 3 presents the three GLMs, with random imputation of missing data, in which we explored the factors associated with mental health-related service use costs among those young people who had used some type of service for their mental health problems. As a result of stage 1 of our two-part model, 84 participants were identified as having positive costs and were, therefore, included in the GLM analyses [34].

**Table 3:** Generalized Linear Models of the associations between cost of services and potential predictors (*n* = 84)

Independent variables	MODEL 1	MODEL 2	MODEL 3
	Coef. (95% CI)	Coef. (95% CI)	Coef. (95% CI)
Young people’s mental health problems			
None	Reference	Reference	Reference
Remittent	− 1.20 (− 4.57 to 2.16)	− 0.60 (− 2.40 to 1.20)	− 0.43 (− 2.21 to 1.36)
Incident	0.01 (− 3.42 to 3.43)	0.33 (− 1.55 to 2.21)	0.60 (− 1.27 to 2.48)
Persistent	0.10 (− 2.40 to 2.59)	− 0.04 (− 1.56 to 1.49)	− 0.11 (− 1.60 to 1.38)
Age (years)			
9–10		Reference	Reference
11–13		0.77 (− 0.41 to 1.95)	0.70 (− 0.41 to 1.81)
14 +		2.38 (0.97 to 3.80)**	2.27 (0.95 to 3.60)**
Gender			
Female		Reference	Reference
Male		0.29 (− 0.14 to 1.65)	0.22 (− 0.60 to 1.05)
Ethnicity			
White		Reference	Reference
Non-white		0.75 (− 0.14 to 1.65)	0.60 (− 0.26 to 1.46)
Maternal education			
Primary school		Reference	Reference
High school		0.53 (− 0.60 to 1.66)	0.50 (− 0.58 to 1.58)
College/university		− 0.18 (− 1.15 to 0.78)	− 0.15 (− 1.06 to 0.76)
Young people’s eligibility for free school meals			
No		Reference	Reference
Yes		1.38 (0.41 to 2.35)**	1.50 (0.58 to 2.43)**
Caregivers’ intended stigma-related behaviour			
Low stigma			Reference
Medium/high stigma			0.56 (− 0.28 to 1.40)

*CI* confidence interval, *dependent variable* cost of using any service, *MODEL 1* dependent variable + child psychopathology, *MODEL 2* MODEL 1 + sociodemographic characteristics, *MODEL 3* MODEL 2 + caregivers’ intended stigma-related behaviour

*p < 0.05

**p < 0.01

In Model 1, which included psychopathology only, no association was found between psychopathology categories and costs. In Model 2, when socio-demographic characteristics (young person’s gender, age and ethnicity, and maternal education) and young person’s eligibility for free school meals (as a proxy for socioeconomic vulnerability) were added to the model, being 14 years of age or older and eligibility for free school meals were associated with higher mental health-related service use costs. In Model 3, when caregivers’ intended stigma-related behaviour was added, being aged 14 years or older and eligibility for free school meals remained associated with higher mental health-related service use costs. When, in Model 3, coefficients were converted into Pound Sterling, costs were, on average, £7598 higher among young people who were aged 14 years or older compared to those aged 9–10 years, and £6713 higher compared to those aged 11–13 years. Young people who were eligible to receive free school meals accrued, on average, had service use costs £5947 greater than those who were not eligible for free school meals.

### Sensitivity analysis

When we compared our logistic regression model with multiple imputation to its equivalent model without imputation, there was an increase in *p* values reported for eligibility for free school meals (from 0.015 to 0.064) and caregivers’ stigma-related behaviour (from 0.040 to 0.091), so that they lost statistical significance in the model not using imputed data. In our GLM models, an increase in the parameter (from 0.75 to 0.97) and a decrease in the *p* value (from 0.100 to 0.045) for ethnicity in Model 2 was observed in the model without multiple imputation. No significant variations were observed in Models 1 and 3. None of the other sensitivity analyses with alternative stigma and ethnicity variables led to changes in the direction and/or statistical significance of the results (data not shown and available from the author).

## Discussion

This study aimed to identify factors which predicted young people’s use of services (and costs) due to mental health problems. Our results show that different factors influence use of services and costs related to service utilisation. Namely, in our analyses, persistent psychopathology, and family socioeconomic disadvantage, represented by young people’s eligibility to receive free school meals, increased the likelihood of service utilisation due to mental health problems. Caregivers’ intended stigma-related behaviour reduced the chance of young people using services. From the three variables associated with service use, only socioeconomic disadvantage was associated with costs. Additionally, young people’s older age (14 years or older) was also associated with costs.

Our results show that persistent psychopathology was associated with greater odds of young people reporting using mental health-related services. However, psychopathology was not associated with costs, which may be considered a proxy measure for intensity of use of services. This suggests that despite increased contact with a variety of services assessed in the study, young people with more persistent psychopathology may fail to receive the level of specialised or intensive care they need. This is substantiated by official figures in the UK which report that, in 2017, less than one-third of children who were referred to CAMHS received treatment 12 months following the referral, whereas another “37% were not accepted into treatment or discharged after an assessment” [3]. That only a small proportion of young people with mental health problems receive help from mental health specialists has also been reported elsewhere. Johnson et al. [37], for example, using data from the 2013–2014 Australian Child and Adolescent Survey of Mental Health and Wellbeing, found that, although over a half of young people with mental health problems had used services in the last 12 months, only 23.9% had contact with a psychologist and 7.1% with a psychiatrist. Moreover, only 11.6% of children with mental health problems had sufficient contact with health professionals to achieve criteria for minimally adequate treatment [38]. That young people with mental health problems should have access to adequate treatment is confirmed by clinical guidelines in the UK, which, for example, usually recommend psychological interventions as the first choice to treat mental disorders among children and adolescents [39–41]. As UK data show [2, 3], even when referred to CAMHS, the majority of young people with mental health problems do not receive any treatment. One hypothesis is that potential flaws may exist in the referral and/or retention of young people with persistent problems to child and adolescent mental health services—Smith et al. [42], for example, have found that children and young people referred to CAMHS in Scotland were more likely to be rejected if they were referred by teachers or if they had emotional and behavioural difficulties rather than common mental health disorder. Further studies aiming to understand such barriers and flaws would inform the implementation of interventions to improve the integration between levels and sectors of care—such interventions have improved general practitioners’ ability to identify and properly refer young people with mental health problems in the Netherlands, for example [43].

In our study, socioeconomic disadvantage was associated both with increased use of services and with higher costs, suggesting that, beyond psychopathology, social problems may be an important driver of service utilisation and costs. This may be related to the fact that young people living under deprived circumstances are disproportionally affected by different types of disability [44], including developmental problems [45], and, therefore, are more likely to use special education provision, as has been shown by Snell et al. [46]. Socioeconomic disadvantage is also associated with other factors, such as stress [47] and a number of psychosocial risk factors [48], such as violence, which may both lead to a psychopathological diagnosis and to sub-clinical mental health problems that may need support.

Finally, whereas age was not associated with use of services, when young people used services, being 14 years of age or older (relative to 9 or 10 years of age) was related to a significant increase in costs. This suggests that adolescents with mental health problems may need more complex interventions than younger children.

As Gronholm et al. [6] had found in the same sample, young people were less likely to use services if their caregiver reported medium/high levels of stigma-related intended behaviours, which is in line with previous findings [49] that stigma leads to families using secrecy and concealment as strategies to deal with mental health problems, thus reducing their likelihood of seeking help. Caregivers’ stigma, nonetheless, was not associated with costs.

## Strengths and limitations

This study provides an up-to-date economic analysis on costs of service use due to mental health problems in a community sample of young people — in contrast to other studies carried out in the UK, such as by Knapp et al. [4], this is the first study looking at the role of stigma as a potential driver of use of services and related costs. The assessment of barriers and facilitators related to use of mental health-related services in a community sample of young people is an important strength, as it can improve our understanding of use of mental health care and associated costs within the population, not limited only to those who have established contact with specific services. In contrast with studies that rely on clinical samples, our community sample allows us to identify differences in the characteristics of young people who do not use services and, hence, provides a more comprehensive picture of barriers to mental health care.

The fact that it is a community sample, however, is also one of the limitations for the purposes of the current analysis. In our sample, a low proportion of participants used services, despite almost half the sample reporting persistent mental health problems, only 84 (20.6%) participants were identified as having positive costs. This limited the statistical power of our GLM models as only the subset of 84 participants who reported some service use were included in the second part of the model. Subsequently, we were not able to run sub-analyses to estimate costs associated with different services and/or sectors, such as health and education, which would have allowed us to make comparisons with other sectoral studies [4, 46]. Moreover, this analysis was based on a convenience sample drawn from collaborating primary schools in Greater London and included only participants who had previously consented to be re-contacted (*n* = 850; 10.5%). Therefore, our sample may not be representative of our study population and is possibly subject to a number of identifiable and unidentifiable biases, which may limit the generalisability of our findings. In our sample, for example, White ethnicity seems to be underrepresented if compared to the UK’s ethnicity profile (87% White), but is comparable to London’s (59.8%), according to UK 2011 census data [50]. The decision to group all non-White ethnic groups into one single category is also an important limitation, resulting in a considerably heterogeneous group. This may explain why, in our analysis, we found no association of use and costs of services with ethnicity, which would be otherwise expected considering that, as according to official figures, black people are the least likely to receive treatment for mental health problems in the UK [51]. Finally, there was a high degree of missing data on key variables, for individuals who were vulnerable and those with severe psychopathology. We were able to minimise potential biases resulting from missingness by running multiple imputation models, which also improved statistical power, particularly in our logistic regression model.

## Conclusion

By showing that variation in mental health service use and related costs is driven by socioeconomic and stigma-related characteristics in addition to psychopathology, our results may have important implications for mental health service delivery, including outreach and referral. In principle, mental health services rely on a positive diagnosis as the main criterion for taking on and keeping patients in care, particularly in relation to eligibility to access specialty care. Our findings suggest that social determinants of mental health problems may also play an important role in the referral, utilisation and inclusion of young people in different types of services for mental health. Integrating mental health into other relevant sectors, such as social care, social support and education is important for increasing access to groups who experience barriers in use and referral to more traditional types of mental health services. School provides an opportunity for universal outreach to young people, and social care services may already be in contact with vulnerable groups who may have more mental health needs. Future research should explore the reasons why children living under socioeconomic disadvantage are more likely to use services and bear higher costs than would be expected based on their psychopathology, and help plan interventions that tackle deprivation and social injustice as part of a wider public health agenda.

That stigma inhibits young people and their families from seeking help shows that investment in anti-stigma programmes is fundamental to increase access to mental health care. Successful examples of such programmes already exist [52] and have proven effective in increasing positive attitudes towards persons with mental health problems and are associated with increased willingness to use services [53].

## Supplementary Information

Below is the link to the electronic supplementary material.

Supplementary file1 (DOCX 18 KB)

## Data Availability

This study reports on data collected as part of the “Child Health and Development Study”, and the associated project “Adolescent Precursors to Psychiatric Disorders: Learning from User Service Engagement”. These data cannot be made publicly available due to ethical restrictions as the consent of participants implied that only the research team will have access to the data provided. Anonymised data from the studies are held by the Principal investigators Dr Sara Evans-Lacko (S.Evans-Lacko@lse.ac.uk) and Dr Kristin R Laurens (Kristin.Laurens@qut.edu.au). Those interested in obtaining these data should contact Drs Evans-Lacko and Laurens to request appropriate approval for access.

## Code availability

All the analyses presented in this manuscript were run using Stata, version 13.1. Those interested in having access to our Stata code should contact the corresponding author.
