# Supplementary material for: Influence of stigma, sociodemographic and clinical characteristics on mental health-related service use and associated costs among young people in the United Kingdom
Source: Eur Child Adolesc Psychiatry. 2022 Jan 27;32(8):1363–73. doi: 10.1007/s00787-022-01947-2 (PMC10326138; doi:10.1007/s00787-022-01947-2)
Supplement: Supplementary file 1 — Supplementary file1 (DOCX 18 KB) [file 787_2022_1947_MOESM1_ESM.docx]

| **Supplemental table 1: Proportion of young people using different types of services due to mental health problems, mean number of nights/visits to services and mean annual costs among young people who used services** | | | | |
| --- | --- | --- | --- | --- |
| Type of service | Number of users | Number of nights/visits* | Unit cost | Annual costs* |
|  | N (%) | Mean (SD) |  | Mean (SD) |
| **General health services** |  |  |  |  |
| Emergency room | 3 (0.7) | 1.0 (0.0) | 206.0 | 206.0 (0.0) |
| Paediatrician | 25 (6.1) | 1.8 (1.5) | 205.0 | 377.2 (299.0) |
| ***Total*** | ***28 (6.9)*** |  |  | ***358.9 (287.8)*** |
| **Mental health services** |  |  |  |  |
| Psychiatric hospital | 2 (0.5) | 45 (21.2) | 620.0 | 27,900.0 (13,152.2) |
| Psychiatric unit | 1 (0.2) | 7 (0.0) | 620.0 | 4,340.0 (0.0) |
| Community mental health centre | 27 (6.6) | 8.8 (6.1) | 236.0 | 2,067.2 (1,452.4) |
| Partial hospitalisation/day care | 1 (0.2) | 130.0 (0.0) | 318.0 | 41,340.0 (0.0) |
| Home therapist, counsellor or family preservation worker | 2 (0.5) | 13.5 (3.5) | 83,0 | 1,120.5 (293.4) |
| Psychologist, psychiatrist or social worker | 13 (3.2) | 4.9 (3.6) | 86.0 | 422.7 (309.0) |
| ***Total*** | ***18 (4.4)*** |  |  | ***6,067.6 (13,633.5)*** |
| **Community services** |  |  |  |  |
| Priest, minister, Rabbi | 3 (0.7) | 1.3 (0.6) | 28.0 | 37.3 (16.2) |
| Acupuncturist or chiropractor | 1 (0.2) | 1 (0.0) | 35.0 | 35.0 (0.0) |
| Self-help group | 14 (3.4) | 10.6 (14.7) | 51.0 | 540.2 (750.1) |
| ***Total*** | ***18 (4.4)*** |  |  | ***428.3 (690.4)*** |
| **Education** |  |  |  |  |
| Special schools/pupil referral units | 8 (2.0) | 4.1 (1.2) | 42.11 | 173.7 (52.5) |
| Special class in regular school | 10 (2.5) | 2.4 (1.6) | 31.58 | 82.8 (47.2) |
| Special help or tutoring in regular class | 15 (3.7) | 66.2 (62.5) | 0.4 | 29.8 (26.1) |
| Counselling in school | 37 (9.1) | 39.2 (20.6) | 0.7 | 29.0 (13.2) |
| ***Total*** | ***48 (11.8)*** |  |  | ***74.3 (82.7)*** |
| **Social care** |  |  |  |  |
| Detention centre | 1 (0.2) | 2 (0.0) | 237.0 | 474 (0.0) |
| Prison or jail | 1 (0.2) | 1 (0.0) | 237.0 | 237.0 (0.0) |
| Probation or juvenile correction officer | 3 (0.7) | 9.3 (10.4) | 230.0 | 2,146.7 (2,393.9) |
| ***Total*** | ***3 (0.7)*** |  |  | ***2,383.2 (2,167.2)*** |
| **TOTAL COSTS** | **84 (20.6)** |  |  | **2,394.4 (7,108.2)** |
| * Mean number of nights/visits and mean costs in the subsample of participants who used services in the past twelve months | | | | |
